# The relationship between mental fatigue awareness and effective decision-making levels of athletes: the role of mediating and moderating variables

**DOI:** 10.1186/s13102-025-01525-4

**Published:** 2026-01-17

**Authors:** Nuriye Şeyma Kara, Mehmet Kara, Ahmet Dönmez, Tuncay Kiratli, Hatice Aslı Çelebioğlu

**Affiliations:** 1 Faculty of Sport Sciences, Hatay Mustafa Kemal University, Hatay, Türkiye Turkey; 2 Faculty of Sport Sciences, Mersin University, Mersin, Türkiye Turkey; 3 Faculty of Sport Sciences, Alanya Alaaddin Keykubat University, Antalya, Türkiye Turkey; 4 Postdoctoral Researcher, Sakarya, Türkiye Turkey; 5 Ministry of National Education, Manisa, Türkiye Turkey

**Keywords:** Fatigue, Decision making, Psychological Well-Being, Gender identity, Sports

## Abstract

This study examined the mediating role of psychological well-being and the moderating roles of gender and sport type (individual vs. team) in the relationship between mental fatigue awareness and intrinsic decision-making in 540 Turkish athletes. Research data were collected online via three different measurement tools. Results indicated a significant indirect effect of mental fatigue awareness on intrinsic decision-making through psychological well-being (indirect effect β = −0.087, *p* < .001), demonstrating partial mediation. Mental fatigue awareness (β = -0.256, *p* < .05) and sport type (β = 0.232, *p* < .05) significantly predicted intrinsic decision-making, with team sport athletes exhibiting higher levels. However, the interaction between mental fatigue awareness and sport type was not a significant predictor (β = 0.021, *p* > .05). Similarly, neither gender (β = 0.083, *p* > .05) nor the interaction between gender and mental fatigue awareness (β = 0.100, *p* > .05) significantly predicted intrinsic decision-making. The study contributes to understanding the interplay of mental fatigue awareness and intrinsic decision-making in athletes, considering the influences of gender and sport type. Future studies should employ longitudinal and experimental designs to clarify causality and to evaluate targeted dual-pronged interventions that address both psychological well-being and mental fatigue management.

## Introduction

Optimal athletic performance arises from a complex interplay of psychological, neurological, affective, and psychomotor factors. While fundamental psychomotor skills remain essential, cognitive and affective elements, including mental fatigue awareness, effective decision-making, and psychological well-being, are increasingly recognized as critical determinants of success. Even with proficient athletic skills, negative mood states and impaired mental functions can significantly hinder performance. Therefore, understanding an athlete’s emotional and mental state is crucial for realistic self-assessment and effective action. Mindfulness, defined by Brown and Ryan [1] as the non-judgmental observation of present moment experiences, including thoughts and feelings, is relevant in this context. Mental fatigue awareness, a specific facet of mindfulness, is the ability to recognize and consciously acknowledge the symptoms of mental fatigue [2]. Kara et al. [3] characterize mental fatigue as an undesirable state leading to reduced focus, procrastination, impaired cause-and-effect reasoning, and performance difficulties. Consequently, an athlete’s level of mental fatigue awareness can significantly impact their decision-making process and overall performance. Greater awareness of mental fatigue may also heighten athletes’ sensitivity to internal cues, which can disrupt the stable cognitive focus required for optimal intrinsic decision-making. Athletes with heightened mental fatigue awareness are better positioned to evaluate decisions realistically, considering their current mental state. This suggests that athletes who are aware of their mental fatigue are potentially better equipped to make sound decisions. The degree of mental fatigue awareness can influence the quality of choices made, which in turn affects both athletic performance and broader life outcomes. Decision-making, in essence, is the ability to select the most suitable option from available alternatives to achieve a desired goal [4]. In this context, recognizing mental fatigue is crucial for effective decision-making, enabling athletes to choose optimal courses of action. The decision-making process involves evaluating information, anticipating possibilities, and analyzing alternatives [5], all of which rely on a healthy mental state. Kahneman’s [6] work highlights that effortful cognitive processes, such as decision-making, draw upon a limited pool of mental resources. This depletion of executive resources directly interferes with intrinsic decision-making, which relies heavily on self-referential evaluation, internal motivation, and reflective cognitive processing. Mental fatigue is understood as a state wherein these resources are depleted, impairing the very executive functions needed for sound judgment. Consequently, as Jia et al. [7] found, individuals experiencing mental fatigue may alter their decision-making processes, often leading to suboptimal outcomes. Smith et al. [8] demonstrated that athletes with higher mental fatigue awareness exhibit greater resilience in challenging situations, leading to more effective strategy development. Psychological well-being, another crucial factor, also influences awareness and decision-making by enhancing coping mechanisms in demanding situations.

Psychological well-being encompasses an individual’s emotional and cognitive satisfaction with life, alongside positive functioning [9]. Dodge et al. [10] define it as the capacity to manage challenges and maintain balance between internal and external resources. Internal resources include resilience and self-efficacy, while external resources encompass environmental conditions and social support [11, 12]. Balancing these resources is vital for stress management and effective decision-making. Awareness plays a key role in strengthening this balance.

Mindfulness facilitates effective resource utilization by promoting the observation of one’s feelings and thoughts [13]. Psychological well-being, by maintaining resource balance, is thus crucial for fostering mindfulness. The relationship between psychological well-being and mindfulness is interactive, enhancing life satisfaction and overall well-being by improving mental and emotional regulation [14]. Baer et al. [15] observed a positive correlation between subjective well-being and mindfulness levels. Keyes [16] found that individuals with higher psychological well-being are more inclined to engage in and benefit from mindfulness practices. Gardner and Moore [17] concluded that mindfulness interventions enhance athletes’ psychological resilience, positively impacting their psychological well-being. Since intrinsic decision-making requires emotional stability, attentional control, and reflective processing, higher psychological well-being provides the cognitive–affective resources necessary to support these processes.

Given that psychological well-being arises from balancing internal and external resources [18], a clear link exists between decision-making and psychological well-being. Kahneman [6] defines decision-making as choosing among alternatives while considering intrinsic and extrinsic factors to achieve a goal. Balanced consideration of these factors is believed to improve decision-making. Imbalances can lead to emotionally driven or emotionally suppressed decisions, potentially hindering a multidimensional perspective and resulting in suboptimal choices. Çetin and Kara [19] define decision-making as a dynamic process of selecting the most appropriate alternative based on personal volition. Their research also highlights the influence of both internal and external factors on athletes’ effective decision-making. Zaki et al. [20] further linked effective decision-making to emotional well-being.

Considering this evidence, modeling the interplay between mental fatigue awareness, effective decision-making levels, and psychological well-being, and examining this model across different variables is warranted. This study aimed to investigate the mediating role of psychological well-being and the moderating roles of sport type and gender in the relationship between mental fatigue awareness and effective intrinsic decision-making levels in athletes. Mediator and moderator analyses are increasingly used to understand variable relationships beyond simple regression analyses [21]. This approach seeks to identify variables that may influence the relationship between dependent and independent variables. A moderator variable alters the strength or direction of the relationship, while a mediator explains the mechanism through which the independent variable affects the dependent variable. Identifying these variables is essential for a comprehensive understanding of variable relationships and for developing targeted interventions. This study aims to contribute to the field by exploring potential intervention strategies focusing on the mediating variable. Examining the moderating effects of gender and sport type aimed to determine how these factors alter the relationship between mental fatigue awareness and decision-making. This research seeks to clarify the complex relationships between these variables.

The current study not only explores this mediation pathway but also investigates how sport type and gender might moderate the outcomes. These factors were chosen based on the theoretical idea that the context of decision-making can influence the effect of an athlete’s internal condition. Contextual demands in sport, such as the level of coordination required or the degree of individual autonomy, influence how athletes perceive and interpret internal cues [12, 17]. Variations in emotional regulation and coping responses—key mechanisms emphasized in psychological functioning—also contribute to differences in decision-related cognitive processing [11, 14]. Therefore, both sport type and gender represent meaningful contextual conditions under which the influence of mental fatigue awareness on intrinsic decision-making may differ. Sport type, whether individual or team, is considered a significant moderator because athletic decision-making is influenced by a complex interaction of internal and external elements [19]. Team sports naturally require athletes to consider more external factors, such as the positions of teammates and opponent strategies, while individual sports focus more on internal signals and self-control. Since mental fatigue can hinder the ability to process complex information [5], it is reasonable to assume that its impact on decision-making might vary depending on whether the main decision-making burden is external or internal. Similarly, gender is examined as a possible moderator. Effective decision-making is strongly associated with emotional well-being and regulation [20], and mindfulness practices are known to improve these aspects [14, 17]. Given that broader psychological research often indicates potential differences in emotional processing and coping mechanisms between genders, it is a valid area of investigation to determine if gender influences the strength of the connection between an athlete’s awareness of their internal state (mental fatigue) and their subsequent decision-making. Based on these theoretical foundations and empirical findings, the present study proposes the following hypotheses to clarify the mechanisms and boundary conditions of the relationship between mental fatigue awareness and intrinsic decision-making.

This research proposes three primary hypotheses: (H_1_) a negative relationship between mental fatigue awareness and intrinsic decision-making; (H_2_) a positive relationship between psychological well-being and decision-making; and (H_3_) a negative relationship between mental fatigue awareness and psychological well-being. Furthermore, the study explores the potential moderating roles of gender and sport type on the mediating effect of psychological well-being (H_4_).

## Materials and methods

This section details the study population and sample, data collection instruments, research procedure, and data analysis methods.

### Research model

This study employed a predictive correlational design, a type of relational model, to investigate the mediating role of psychological well-being and the moderating effects of sport type and gender on the relationship between mental fatigue awareness and decision-making in athletes. Predictive correlational models are used to examine relationships between variables and determine the influence of independent variables on dependent variables [22]. TPrior to testing the structural relationships, the adequacy of the measurement model was evaluated to ensure that each construct was reliably represented in the SEM framework.

### Study group

The study population comprised active, licensed athletes engaged in sports across Turkey. Participants were recruited through non-probability sampling methods, specifically convenience and criterion sampling, based on voluntary participation. The sample included athletes competing at various levels: local/regional (45%), national (40%), and international (15%). These competitive levels correspond to amateur (local/regional), semi-professional (national), and elite (international) classifications commonly used in sport science research. A total of 540 athletes, 312 women (57.7%, x̄_year_ = 21.90 ± 6.45) and 228 men (42.3%, x̄_year_ = 23.96 ± 8.67) who met the criteria and provided complete data were included in the analysis. While this sampling strategy facilitated recruitment, its limitations regarding generalizability are acknowledged in the discussion section. Research data were collected online via Google Forms in 20/01/2025–17/03/2025. Table 1 summarizes participant demographics.

**Table 1:** Demographic characteristics of athletes by age, sports experience, and gender

				Sports Experience			
				1–5 Years	6–10 Years	11 + Years	Total
Age 18 and Under	Gender	Women	% Gender	33 50.8%	9 13.8%	23 35.4%	65 100.0%
		Men	% Gender	13 33.3%	10 25.6%	16 41.1%	39 100.0%
	Total			46 44.2%	19 18.3%	39 37.5%	104 100.0%
Age 19–25	Gender	Women	% Gender	111 52.9%	37 17.6%	62 29.5%	210 100.0%
		Men	% Gender	33 25.6%	38 29.5%	58 44.9%	129 100.0%
	Total			144 42.5%	75 22.1%	120 35.4%	339 100.0%
Athletes 26+	Gender	Women	% Gender	20 54.1%	7 18.9%	10 27.0%	37 100.0%
		Men	% Gender	18 30.0%	13 21.7%	29 48.3%	60 100.0%
	Total			38 39.2%	20 20.6%	39 40.2%	97 100.0%
Total	Gender	Women	% Gender	164 52.6%	53 17.0%	95 30.5%	312 100.0%
		Men	% Gender	64 28.1%	61 28.6%	103 45.2%	228 100.0%
	Total			228 42.2%	114 21.1%	198 36.7%	540 100.0%

Table 1 details the demographic breakdown of participants. Of the 540 participants, 57.7% were women and 42.3% were men. Age groups were distributed as follows: 19.3% were 18 years or younger, 62.8% were between 19 and 25 years, and 17.9% were 26 years or older. In terms of sports experience, 42.2% had 1–5 years, 21.1% had 6–10 years, and 36.7% had 11 years or more of licensed sports experience.

### Ethical approval

Approval for the study was granted by the Hatay Mustafa Kemal University Ethics Committee (10/01/2025, number 1/4), in compliance with the Ethical Standards of the 2024 Helsinki Declaration.

### Data collection tools

#### The scale of effective Decision-Making in sport (SEDMS)

Developed by Çetin and Kara [19], this 15-item scale measures decision-making in sports across two sub-dimensions: Intrinsic and Extrinsic Decision-Making. It uses a 5-point Likert scale (1 = Strongly Disagree to 5 = Strongly Agree). The Intrinsic Decision-Making sub-dimension (7 items) and the Extrinsic Decision-Making sub-dimension (8 items) demonstrated good internal consistency (Cronbach’s α = 0.85 and 0.87, respectively). The two factors explained 54% of the variance in the original validation study. This study utilized only the Intrinsic Decision-Making sub-dimension due to its theoretical relevance to the research model. In the current study, Cronbach’s α for the Intrinsic Decision-Making sub-dimension was 0.88.

#### Mental Fatigue Awareness Scale in Athletes (MFASA)

Developed by Kara et al. [3], this 25-item, single-factor scale measures an athlete’s awareness of mental fatigue symptoms. Items are rated on a 5-point Likert scale (1 = Never to 5 = Always). The scale explained 52.486% of the variance and demonstrated high internal consistency (Cronbach’s α = 0.96) in its development study. In this study, Cronbach’s α was 0.96.

#### Psychological Well-Being Scale (PWBS)

Developed by Diener et al. [23] and adapted to Turkish by Telef [24], this 8-item, single-dimension scale measures psychological well-being on a 7-point Likert scale (1 = Strongly Disagree to 7 = Strongly Agree). The scale showed good internal consistency (Cronbach’s α = 0.87) and explained 53% of the variance in the Turkish adaptation. In this study, Cronbach’s α was 0.89. The PWBS has been widely validated across different populations, supporting its suitability for use in athlete samples.

Reliability analyses for the current study confirmed satisfactory internal consistency for all scales: Intrinsic Decision-Making sub-dimension (α = 0.88), Mental Fatigue Awareness Scale (α = 0.96), and Psychological Well-Being Scale (α = 0.89).

### Data analysis

Structural Equation Modeling (SEM) was used to examine the mediating and moderating hypotheses on the open-source Jamovi statistical software. Initial data collection yielded 648 responses. Prior to analysis, data were screened for outliers, missing values, and assumption violations. Assumption testing led to the exclusion of 108 cases, resulting in a final sample of 540 observations. No missing data were detected. Assumption testing, analogous to multiple regression diagnostics, included normality, outlier identification (univariate and multivariate), multicollinearity, and autocorrelation of errors.

Normality was evaluated by comparing median, mode, and mean values, which were sufficiently similar to indicate an acceptable distribution. Skewness and kurtosis values were also examined, with standardized skewness values ranging from − 0.967 to 0.849 and kurtosis values ranging from − 1.436 to 1.229. These values fall within the ± 1.5 range, considered acceptable for assuming normality according to George and Mallery [25].

#### Outlier detection and treatment

Outliers were assessed via Mahalanobis distances for multivariate outliers, and Z-scores and box plots/scatter plots for univariate outliers. All Z-scores were within ± 3.11, below the Tabachnick criteria (± 4), indicating no univariate outliers. For multivariate outliers, Mahalanobis distances were calculated and compared to the chi-square distribution (χ² with degrees of freedom equal to the number of variables, *p* <.001). Twenty-six cases exceeding the critical value (χ²(40, 0.001) = 73.402) were removed. Box plot and scatter plot analyses further identified and removed 72 univariate outliers, yielding the final sample of 540 participants. These steps ensured that only athletes whose data met statistical assumptions were retained in the final dataset.

#### Assumption testing

Autocorrelation of errors was assessed through the Durbin-Watson (D-W) statistic, which was 1.78, falling within the acceptable range (approximately 1.5 to 2.5), indicating independence of errors [26]. Multicollinearity was examined by Variance Inflation Factor (VIF) and tolerance values. VIF values ranged from 1.86 to 3.73, and tolerance values ranged from 0.273 to 0.609. Given that VIF values were below 5 and tolerance values were above 0.20 [27, 28], multicollinearity was not considered a problem.

#### Mediation analysis

**Fig. 1:**
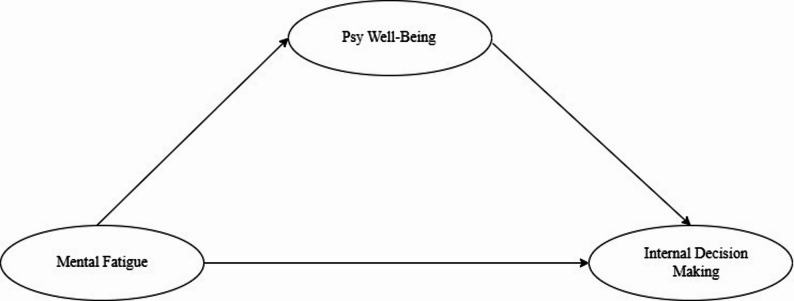
Mediation model

Mediation analysis followed the procedures outlined by Baron and Kenny [29]. First, the direct effect of mental fatigue awareness (independent variable) on intrinsic decision-making (dependent variable) was examined. Then, psychological well-being (mediator variable) was introduced into the model to assess its mediating role. A significant reduction in the direct effect with the mediator in the model, while the indirect effect remains significant, indicates mediation. Full mediation occurs when the direct effect becomes non-significant, and partial mediation occurs when the direct effect remains significant but is reduced. Figure 1 illustrates the mediation model.

Figure 1 presents a research model examining the interrelationships between mental fatigue, psychological well-being, and decision-making in sports. Mental fatigue represents a negative state of mental capacity that impairs individuals’ ability to perform daily activities at optimal levels [3] and leads to decreased cognitive performance [30, 31]. This impairment manifests through alterations in reasoning processes, behavioral patterns, and physiological responses during decision-making [32]. Besides, Jia et al. [7] demonstrate that mental fatigue can significantly distort decision-making processes, resulting in suboptimal choices. The relationship between these variables appears to operate through multiple pathways. Decision-making represents a process that requires minimizing factors such as rushed judgment, pressure, and panic responses [19]. Mental fatigue can compromise this process both directly and indirectly. Research indicates that mental fatigue diminishes psychological well-being [33], particularly through its impact on emotion regulation capacity [34], suggesting a robust relationship between these constructs.

Furthermore, psychological well-being emerges as a crucial factor in decision-making processes. Recent empirical evidence supports this connection: Joshanloo [35] demonstrated that individuals with higher levels of psychological well-being tend to make more positive decisions, while Lasyena et al. [36] found that decision quality and decision-making behaviors are particularly sensitive to psychological factors, including stress and anxiety levels. This theoretical framework, supported by the empirical evidence, provides the foundation for the study’s hypotheses, suggesting a complex interplay between mental fatigue, psychological well-being, and decision-making effectiveness in athletic contexts.

- H_1_: Mental fatigue has a significant negative effect on intrinsic decision-making.
- H_2_: Mental fatigue has a significant negative effect on psychological well-being.
- H_3_: Psychological well-being has a significant positive effect on intrinsic decision-making.
- H_4_: Psychological well-being mediates the negative effect of mental fatigue on intrinsic decision-making.

**Fig. 2:**
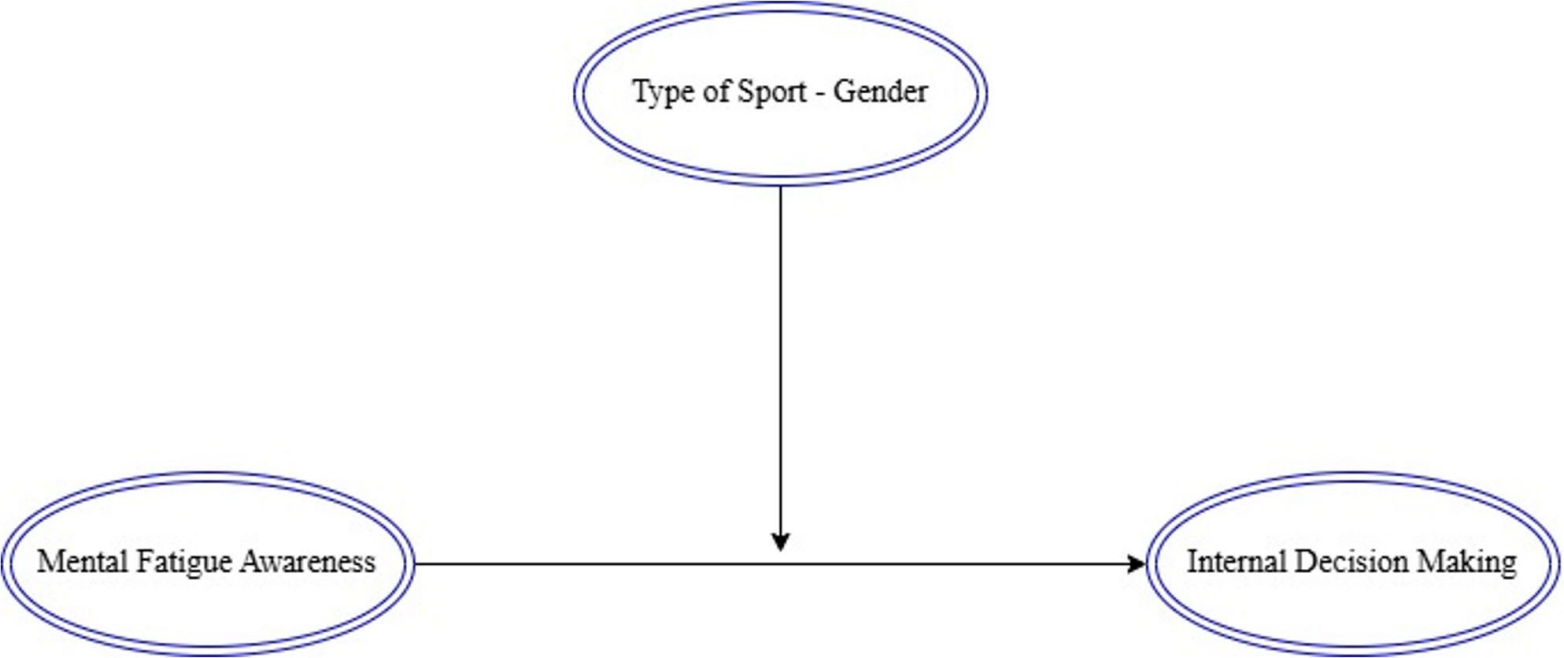
Moderation model

After confirming adequate model fit in the measurement model, structural paths were examined to test the mediation and moderation hypotheses. The moderation analysis examined how sport type and gender influenced the relationship between the primary variables. Moderation was assessed by examining the statistical significance of the interaction term (independent variable × moderator variable). The statistical significance of these interaction terms served as the criterion for establishing moderation effects. Specifically, a significant interaction term indicated the presence of a moderating effect, suggesting that the relationship between the independent and dependent variables varied across different levels of the moderator. Figure 2 illustrates the moderation model testing how both sport type and gender moderate the primary relationships in the study.

The following hypotheses were tested within the moderation model:

- H_5_: Sport type moderates the effect of mental fatigue on intrinsic decision-making
- H_6_: Gender moderates the effect of mental fatigue on intrinsic decision-making.

Beyond the standard assumptions for multivariate statistical analyses, the study’s mediation and moderation analyses required two additional prerequisites. Most critically, prior to testing the structural model for mediation effects, it was essential to validate the measurement model’s adequacy. This validation ensures that the measurement components appropriately represent the constructs before examining their structural relationships. Consequently, we first conducted measurement model testing within the Structural Equation Modeling (SEM) framework. Table 2 presents the detailed findings from these measurement model analyses.

**Table 2:** Confirmatory factor analysis results of the scales

	CMIN/DF (x^2^/df)	CFI	GFI	NFI	RMR	RMSEA	Cronbach’s α
Intrinsic Decision-Making	104/24 = 4.33	0.97	0.95	0.97	0.033	0.084	0.88
Mental Fatigue Awareness Scale in Athletes	2043/495 = 4.12	0.96	0.84	0.96	0.055	0.080	0.96
Psychological Well-Being Scale	92.37/20 = 4.61	0.98	0.96	0.97	0.035	0.085	0.89

Table 2 demonstrates that all scales exhibited satisfactory fit indices, supporting the measurement models’ validity. The fit statistics for the Intrinsic Decision-Making were χ²/df = 4.33, CFI = 0.97, GFI = 0.95, NFI = 0.97, RMR = 0.033, RMSEA = 0.084, and Cronbach’s α = 0.88. For the Mental Fatigue Awareness, χ²/df = 4.12, CFI = 0.96, GFI = 0.84, NFI = 0.96, RMR = 0.055, RMSEA = 0.08, and Cronbach’s α = 0.96. For the Psychological Well-Being scale, χ²/df = 4.61, CFI = 0.98, GFI = 0.96, NFI = 0.97, RMR = 0.035, RMSEA = 0.085, and Cronbach’s α = 0.89. These results verify the measurement model’s adequacy within the structural equation modeling framework, indicating that the research findings are based on reliable measurements. The second crucial prerequisite concerns the relationships between independent, dependent, and mediator variables. Table 3 presents these relationships’ significance levels and magnitude.

**Table 3:** Correlation analysis results for factors and scales

*N* = 540	X̄	SD	1	2	3
1. Intrinsic Decision Making	3.8508	0.6649	1	− 0.286**	0.296**
2. Mental Fatigue Awareness	2.3318	0.7219		1	− 0.457**
3. Psychological Well-Being	5.1609	1.2505			1

**p <.01, * *p* <.05

Table 3 shows significant correlations between all variables. Intrinsic decision-making showed a significant negative correlation with mental fatigue awareness (*r* = −.286, *p* <.01) and a significant positive correlation with psychological well-being (*r* =.296, *p* <.01). Mental fatigue awareness and psychological well-being showed a significant negative correlation (*r* = −.457, *p* <.01). The correlation results provided preliminary support for H1, H2, and H3, showing that mental fatigue awareness was negatively associated with intrinsic decision-making and psychological well-being, while psychological well-being showed a positive correlation with intrinsic decision-making.

Mediation and moderation are distinct statistical concepts describing how a third variable influences the relationship between an independent variable (X) and a dependent variable (Y). Mediation explains how X affects Y through a mediating variable (M), while moderation explains when X affects Y through a moderating variable (Z). According to Baron and Kenny (1986), assessing mediation involves several key steps. First, a significant relationship between X and Y (X → Y) must be established. Second, X must significantly predict M (X → M). Third, M must significantly predict Y when controlling for X (M → Y | X). Finally, the effect of X on Y should be reduced when M is introduced into the model. If this effect becomes non-significant, it suggests full mediation; if it remains significant but smaller, it suggests partial mediation. While the Baron and Kenny approach is widely recognized, contemporary practice often emphasizes directly assessing the indirect effect (X → M → Y) through methods like bootstrapping, rather than solely relying on the reduction of the X → Y path. Moderation, conversely, focuses on how the strength or direction of the X → Y relationship changes depending on the level of Z. While a significant X → Y relationship may exist, it is not a requirement for moderation. The defining feature of moderation is a significant interaction between X and Z (X × Z), indicating that the effect of X on Y varies across different values of Z. This interaction can strengthen, weaken, or even reverse the relationship between X and Y. The significance of the X × Z product term is the primary evidence for a moderating effect.

## Findings

### Findings related to the mediation model

**Fig. 3:**
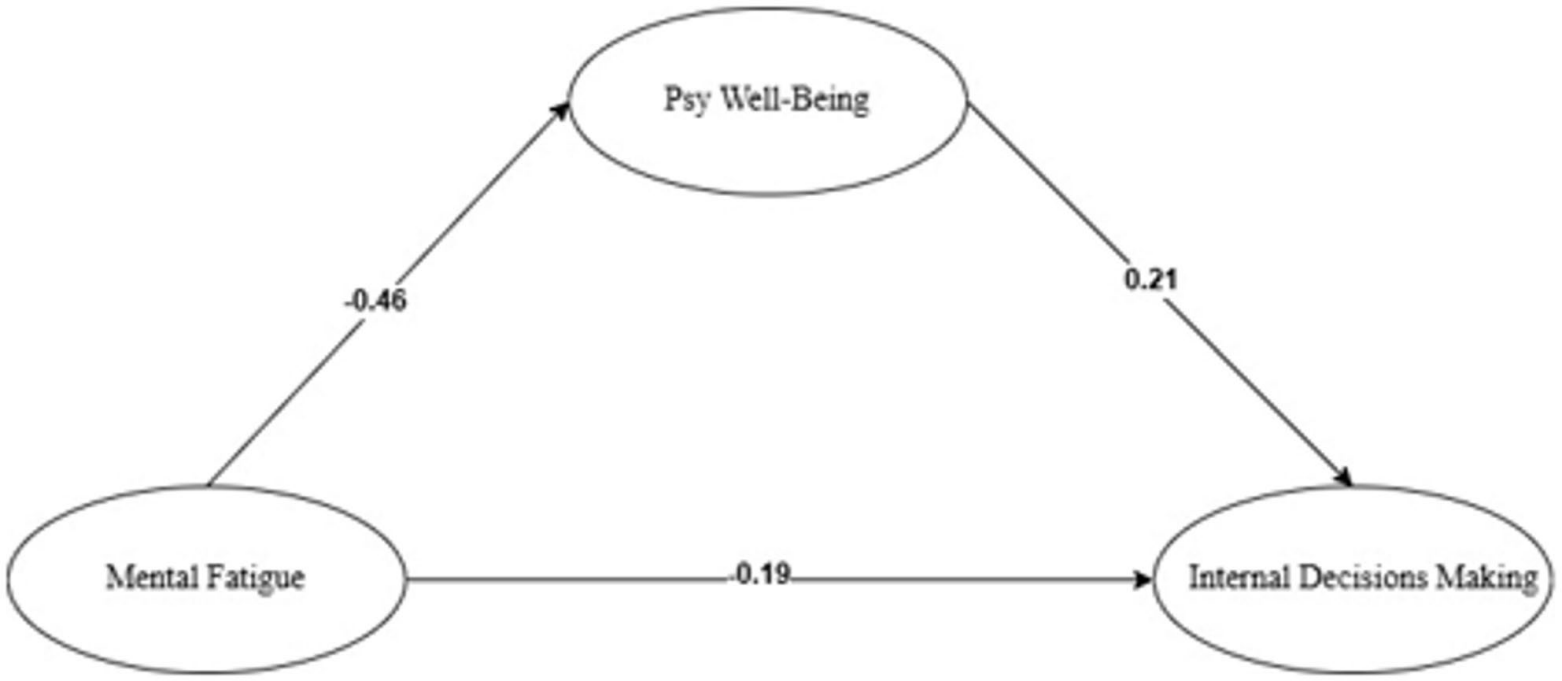
Research model diagram and beta coefficients

The mediation model examining the mediating role of psychological well-being in the relationship between mental fatigue awareness and intrinsic decision-making was tested via SEM on Jamovi software. Figure 3 presents the path diagram with beta coefficients.

To understand the causal relationships, both direct and indirect effects were analyzed. Direct effect refers to the impact of the independent variable on the dependent variable without mediation. Indirect effect, or mediation effect, occurs when the mediator variable explains the relationship between the independent and dependent variables. Table 4 summarizes the mediation effect analysis.

**Table 4:** Mediation effect analysis of the model

	Estimate	SH	β	Z	*p*	Mediation Effect (%)
Mental Fatigue◊ Psy_Well-Being	−0.791	0.0663	−0.456	−11.94	< 0.001	
Psy_Well-Being ◊Int_Decision-Making	0.111	0.0242	0.208	4.58	< 0.001	
Mental Fatigue◊ Int_Decision-Making	−0.176	0.0419	−0.191	−4.21	< 0.001	
Indirect Effect	−0.087	0.0205	−0.095	−4.28	< 0.001	33.2
Direct Effect	−0.176	0.0419	−0.191	−4.21	< 0.001	66.8
Total Effect	−0.263	0.0380	−0.286	−6.95	< 0.001	100.0

According to Table 4, the total effect of mental fatigue awareness on intrinsic decision-making is negative and significant (β = − 0.286; *p* <.05), supporting hypothesis H_1_. Mental fatigue awareness had a significant negative effect on psychological well-being (β = − 0.456; *p* <.05), psychological well-being had a significant positive effect on intrinsic decision-making (β = 0.208; *p* <.05), and mental fatigue awareness had a significant negative direct effect on intrinsic decision-making (β = − 0.191; *p* <.05). These findings appear to support hypotheses H_2_ and H_3_.

Including psychological well-being as a mediator reduced the magnitude of the direct effect of mental fatigue awareness on intrinsic decision-making (from β = − 0.286 to β = − 0.191). This reduction, represented by the significant indirect effect (β = − 0.095; *p* <.001), indicates partial mediation by psychological well-being. The indirect effect accounts for 33.2% of the total effect, and the direct effect accounts for 66.8%, thus confirming hypothesis H_4_.

### Moderator role findings

Table 5 presents the results of the moderation analysis for sport type.

**Table 5:** Moderating role of sport type variable in the relationship between mental fatigue and decision-making in sports

			%95 Confidence Interval			
	Estimate	SE	Lower	Upper	Z	*p*
Mental Fatigue	−0.2560	0.0381	−0.333	−0.178	−6.714	< 0.001
Sport TYPE	0.2323	0.0542	0.123	0.341	4.283	< 0.001
Mental_Fatigue x TYPE	0.0208	0.0818	−0.153	0.192	0.255	0.799

**Fig. 4:**
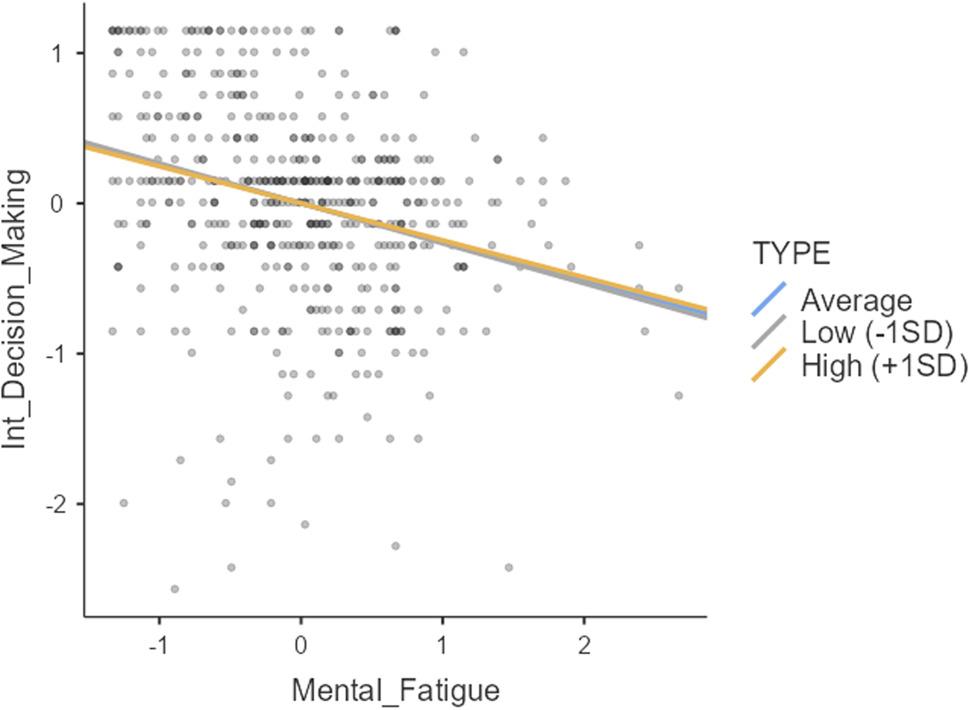
Moderating effect of sport type

The results presented in Table 5 indicate that both mental fatigue awareness and sport type (individual vs. team) significantly predicted intrinsic decision-making (β = − 0.2560; *p* <.001; β = 0.2323; *p* <.001, respectively). However, the interaction effect between mental fatigue awareness and sport type was not a significant predictor of intrinsic decision-making (β = 0.0208; *p* >.05). This suggests that sport type does not moderate the relationship between mental fatigue awareness and intrinsic decision-making. Figure 4 visually represents this lack of moderation.

Figure 4 illustrates that regardless of sport type, the relationship between mental fatigue awareness and intrinsic decision-making remains consistent across low, medium, and high levels of mental fatigue awareness. The negative predictive effect of mental fatigue awareness on intrinsic decision-making is evident for both individual and team sport athletes. Table 6 presents the results of the moderation analysis for gender.

**Table 6:** Moderating role of gender variable in the relationship between mental fatigue and decision making in sports

			%95 Confidence Interval			
	Estimate	SE	Lower	Upper	Z	*p*
Mental Fatigue	−0.2524	0.0380	−0.3309	−0.181	−6.63	< 0.001
Gender	0.0832	0.0554	−0.0255	0.190	1.50	0.133
Mental_Fatigue x Gender	0.0997	0.0813	−0.0698	0.251	1.23	0.220

**Fig. 5:**
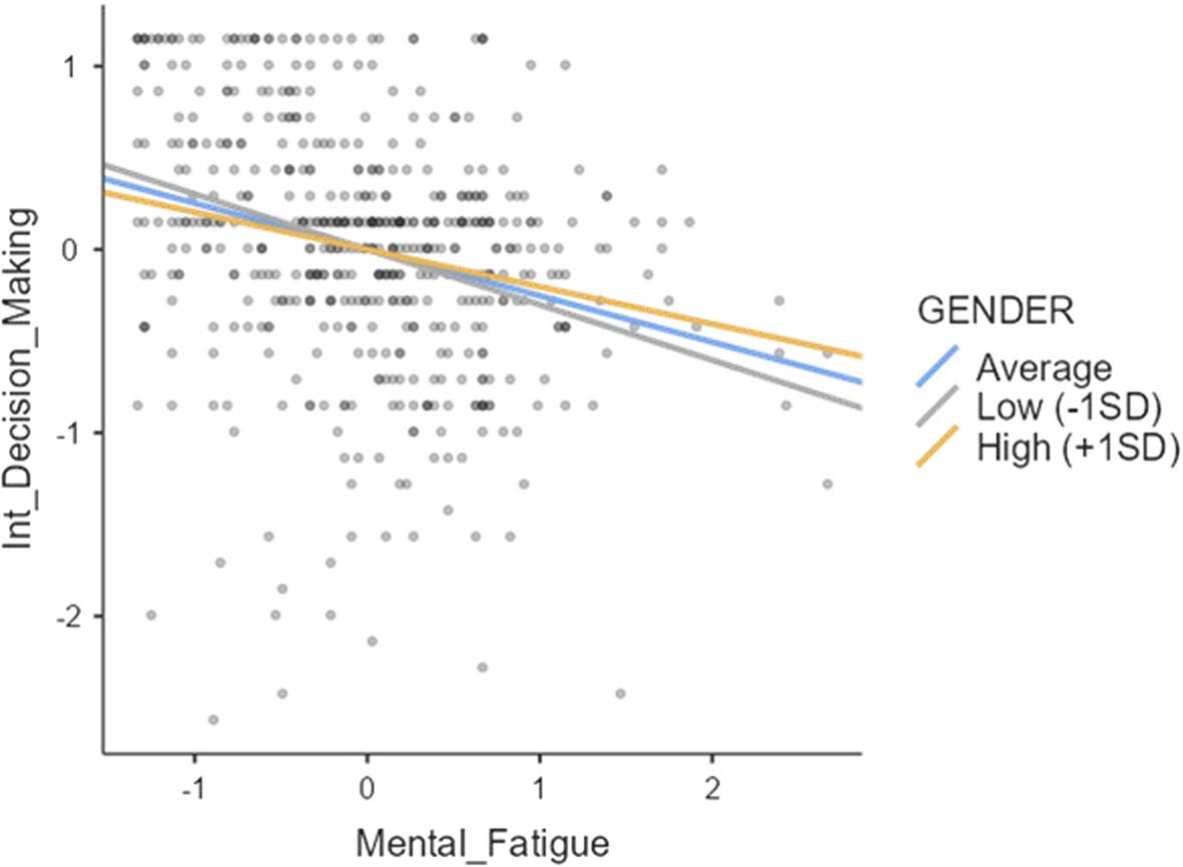
Moderating effect of gender

Table 6 shows that neither gender (β =.0832; p >.05) nor the interaction effect between gender and mental fatigue awareness (β =.0997; p >.05) significantly predicted intrinsic decision-making. Therefore, hypothesis H6, proposing a moderating effect of gender, was rejected. Similar to the sport type moderation analysis, the primary finding is the consistent negative predictive relationship between mental fatigue awareness and intrinsic decision-making, irrespective of gender. (Fig 5) visually represents this.

Figure 5 demonstrates that the negative predictive relationship between mental fatigue awareness and intrinsic decision-making holds true for both men and women athletes across varying levels of mental fatigue awareness.

## Discussion and Conclusion

This study examined the complex interplay between mental fatigue awareness, intrinsic decision-making, and psychological well-being in athletic performance, while also investigating how gender and sport type might moderate these relationships. The overall pattern of results supports the core assumptions of cognitive resource theories, which suggest that increased attention to internal fatigue cues draws on the same limited cognitive resources required for effective decision-making. The correlation analyses revealed several significant patterns that illuminate our understanding of these psychological dynamics. These patterns lay the foundation for understanding both the direct and indirect mechanisms through which mental fatigue awareness influences decision-making performance. First, we found a negative relationship between intrinsic decision-making and mental fatigue awareness, suggesting that athletes who are more acutely aware of their mental fatigue tend to exhibit decreased for intrinsic decision-making. Second, the analysis revealed a positive association between intrinsic decision-making and psychological well-being, indicating that athletes with higher levels of psychological well-being demonstrate enhanced intrinsic decision-making capabilities. Finally, we observed a negative correlation between mental fatigue awareness and psychological well-being, suggesting that heightened awareness of mental fatigue corresponds with diminished psychological well-being. This relationship is consistent with cognitive depletion theories, which argue that increased monitoring of internal fatigue cues consumes attentional resources and contributes to reduced emotional stability. These findings collectively paint a picture of interconnected psychological processes where mental fatigue awareness may impact decision-making both directly and through its influence on psychological well-being, highlighting the importance of considering these factors holistically in athletic performance contexts. This dual influence highlights the need to interpret mental fatigue awareness not only as a performance-related factor but also as a psychological construct capable of shaping emotional states and regulatory capacities.

Intrinsic decision-making, driven by personal feelings and inner motives, might lead to decreased awareness of mental fatigue [37]. However, from a cognitive load perspective, reduced awareness of internal states may limit an athlete’s capacity to recognize early signs of resource depletion, making intrinsic decision-making vulnerable under mentally demanding conditions. Athletes prioritizing emotional responses might be less attuned to their mental fatigue state. This reduced attunement may have short-term functional benefits, such as maintaining motivation, but may also obscure the early emergence of cognitive exhaustion that undermines future decision quality. For instance, an athlete focused on immediate emotional reactions after losses might overlook the underlying mental fatigue contributing to those losses. Such patterns are consistent with theories suggesting that emotional immediacy can overshadow internal monitoring processes, particularly when athletes rely heavily on affect-driven decision frameworks. Consistent with this, Balconi et al. [38] found that increased mental fatigue impairs attention and information processing. These attentional disruptions provide a plausible mechanism explaining why intrinsic decision-making may decline when athletes fail to detect or correctly interpret signs of cognitive strain. Russell et al. [39] also reported that mental fatigue negatively affects athletes’ situational assessment during games. Taken together, these findings indicate that the interaction between emotional prioritization and reduced fatigue awareness may jointly impair the cognitive evaluations required for high-quality intrinsic decisions.

Conversely, prioritizing emotional processes in intrinsic decision-making can positively influence psychological well-being. Acting in accordance with one’s emotions can enhance well-being by aligning actions with desires. In sports, even if emotionally driven decisions lead to suboptimal outcomes, the act of behaving in accordance with one’s feelings can contribute to a sense of psychological well-being. Mouratidis and Michou [40] found that intrinsic motivation, closely linked to emotional drivers, helps athletes cope with stress and fosters positive perceptions, thereby increasing psychological resilience.

Mental fatigue awareness, defined as recognizing mental fatigue symptoms [3, 38], is inherently linked to emotional awareness and psychological well-being. However, heightened emotional awareness, particularly of negative states like mental fatigue, might paradoxically decrease immediate psychological well-being. Focusing on fatigue symptoms could shift attention towards negative emotions, potentially diminishing overall well-being. Counter to this, Cohen et al. [41] found that recognizing mental fatigue symptoms actually increases life satisfaction, possibly through proactive coping strategies. In the context of sports, an athlete highly aware of mental fatigue might experience reduced psychological well-being if this awareness primarily focuses on negative feelings and potential performance decrements. Abbott et al. [42] suggested that mental fatigue in athletes can lead to decreased motivation, negatively impacting psychological well-being.

The mediation model findings revealed significant relationships among mental fatigue awareness, psychological well-being, and decision-making. Specifically, awareness of mental fatigue negatively predicted psychological well-being (β = − 0.46), while psychological well-being positively predicted intrinsic decision-making (β = 0.21). Additionally, awareness of mental fatigue had a direct negative effect on intrinsic decision-making (β = − 0.19). These results suggest that heightened awareness of mental fatigue can detrimentally impact psychological well-being, potentially by amplifying emotional sensitivity. Individuals who focus excessively on their internal states, such as mood and fatigue, may experience diminished psychological well-being, as they become more attuned to their emotional and cognitive burdens. This aligns with the findings of Rouse et al. [43], who demonstrated that perceived mental fatigue is associated with lower levels of psychological well-being. Nevertheless, this adverse connection might only reflect a short-term impact. In the long run, this awareness, though initially unsettling, can be seen as a vital precursor for adaptive self-regulation, which is a fundamental aspect of self-determination theory [44]. By identifying the signs of mental fatigue, athletes gain the ability to employ proactive coping strategies, such as planned rest or specific mindfulness practices, which have been proven to boost psychological resilience [17]. This approach of recognizing and tackling challenges is crucial for nurturing resilience and well-being over time [11]. Consequently, being aware of mental fatigue could serve as a “double-edged sword”: linked to reduced well-being in the immediate term, but crucial for developing the coping skills that lead to improved psychological well-being in the future.

In the context of sports, athletes who are acutely aware of their mental fatigue—often resulting from rigorous training regimens—may experience a decline in motivation, which can further exacerbate psychological distress. This decline in motivation may not only hinder their engagement in future training but also negatively affect their overall psychological well-being. Deci and Ryan [44], prominent theorists in motivation research, distinguish between intrinsic and extrinsic motivation, noting that intrinsic motivation is driven by internal emotions and personal satisfaction, whereas extrinsic motivation is influenced by external rewards or pressures. Given that mental fatigue awareness is closely tied to intrinsic motivation, its negative impact on psychological well-being becomes evident. When athletes become overly aware of their mental fatigue, they may focus excessively on their emotional state, leading to a reduction in intrinsic motivation and, consequently, a decline in psychological well-being. This interplay underscores the importance of managing mental fatigue awareness to preserve both motivation and psychological health in athletic performance.

The finding that psychological well-being partially mediates the relationship between mental fatigue awareness and intrinsic decision-making suggests that while psychological well-being can buffer the negative effects of mental fatigue awareness on decision-making, it does not entirely eliminate them. Psychological well-being, encompassing positive emotions and a sense of meaning in life [45], may foster a more positive approach to mental fatigue awareness. Athletes with higher psychological well-being may be better equipped to use positive self-talk and cognitive reappraisal to manage mental fatigue and its impact on decision-making. For example, an athlete aware of mental fatigue from training might use self-reassurance to mitigate negative feelings and maintain focus. Seligman’s [46] model of psychological well-being emphasizes the role of positive emotions in enhancing cognitive and affective processes, including decision-making.

The findings regarding the moderating role of mental fatigue and sport type revealed that both factors independently and significantly predicted effective decision-making in sport. However, the interaction effect between mental fatigue and sport type did not emerge as a statistically significant predictor of decision-making. This suggests that while mental fatigue and sport type each exert an independent influence on effective decision-making, their combined effect does not significantly alter this relationship. In other words, sport type moderates the relationship between mental fatigue and effective decision-making, but this moderating effect is not strong enough to be statistically significant. These results highlight that mental fatigue awareness is a critical factor that can influence decision-making processes in sports. Specifically, heightened awareness of mental fatigue may impair an individual’s ability to make effective decisions, as it can introduce external cognitive and emotional burdens that interfere with the decision-making process.

Similarly, sport type itself, however, does influence decision-making. Individual and team sports demand different cognitive and social skills in decision-making [47]. Team sports emphasize strategic thinking, communication, and cooperation, enabling rapid, coordinated decisions. Individual sports rely more on self-motivation and discipline for independent decision-making [48]. Despite these differences, the core cognitive resources (attention, memory, decision-making processes) are crucial in both sport types [49]. Mental fatigue awareness can deplete these resources regardless of the specific demands of individual or team sports. Thus, sport type does not appear to moderate the impact of mental fatigue on decision-making.

Our hypothesis that gender would influence the link between awareness of mental fatigue and decision-making (H6) was not confirmed. We had anticipated that gender-related differences in emotional processing and coping mechanisms, which are associated with decision-making quality [20], might play a role. However, the lack of support for this hypothesis indicates that, similar to the impact of sport type, the fundamental cognitive impairment caused by mental fatigue might be strong enough to override any gender-based differences in emotional regulation. Although men and women might use different methods to handle stress or fatigue over time, the immediate effect of acute mental fatigue on decision-making seems to be uniform. This underscores the significant impact of mental fatigue on the essential cognitive framework that supports effective decision-making in high-pressure sports settings.

Mental fatigue awareness, defined as the recognition of fatigue and the ability to manage it [50], can significantly impact decision-making processes. However, psychological well-being, which is associated with the capacity to manage negative emotions [51], may serve as a protective factor that mitigates the adverse effects of mental fatigue awareness. According to Ryan and Deci [52], psychological well-being fosters resilience and emotional regulation, which could help athletes cope with the cognitive and emotional challenges posed by mental fatigue. The study identified psychological well-being as a partial mediator in the relationship between mental fatigue awareness and effective decision-making, indicating that while mental fatigue awareness directly affects decision-making, psychological well-being also plays a compensatory role in this dynamic.

### Limitations and future directions

This study has several limitations that should be recognized, which also suggest directions for future research. Firstly, the study’s cross-sectional design prevents us from establishing causal links between the variables. While our model is theoretically based, longitudinal or experimental studies are necessary to verify the direction of the observed effects. Secondly, our results are based solely on self-reported data, which might be influenced by social desirability bias. Future research could improve by including objective performance metrics or physiological indicators of mental fatigue. Lastly, participants were selected through convenience sampling. Although this approach was practical, it might restrict the applicability of our findings to the wider athlete population. Future studies should strive to employ more varied and representative sampling methods to confirm these findings across different groups of athletes. Specifically, this limitation means that the stability of the identified moderated mediation model cannot be guaranteed across different athletic subgroups (e.g., elite vs. amateur), making such replication crucial.

## Recommendations

Based on the findings of this research, the following recommendations are offered to guide future investigations and inform applied practice:

- Based on our central finding that psychological well-being partially mediates the relationship between mental fatigue awareness and decision-making. Future research should develop and evaluate targeted intervention programs designed to enhance athletes’ psychological well-being. Such programs could incorporate techniques such as mindfulness training, positive psychology interventions, or emotion regulation skills. Studies could then examine the impact of these interventions on both psychological well-being and decision-making processes in sport. Crucially, because this mediation was only partial, such research should also investigate whether a dual-pronged approach—targeting both well-being and fatigue management directly—is most effective.
- Given the significant direct and indirect negative effects of mental fatigue awareness identified in our model, research should explore strategies to improve athletes’ ability to recognize and manage mental fatigue. This could involve investigating the effectiveness of planned rest protocols, mental training techniques, or other interventions aimed at increasing and controlling mental fatigue awareness.
- This study focused on the mediating role of psychological well-being. Future research could expand this investigation by examining other potential mediating variables, such as self-efficacy, intrinsic motivation, stress coping skills, or resilience, in the relationship between mental fatigue awareness and effective decision-making.
- The current study provides a cross-sectional snapshot. Longitudinal research designs are needed to examine how the mediating effect of psychological well-being, and other potential mediators, changes over time, particularly during periods of intense training and competition.
- While this study identified a correlational relationship, future research should employ experimental designs to more rigorously test the causal mediating role of psychological well-being. This could involve implementing interventions to improve psychological well-being and then assessing the impact on both mental fatigue awareness and decision-making, thereby directly testing the hypothesized mediation pathway. Such designs would provide stronger evidence for the causal link between these variables.

## Data Availability

The datasets generated during and/or analyzed during the current study are available from the corresponding author on reasonable request.
